# Exploring the overlapping binding sites of ifenprodil and EVT‐101 in GluN2B‐containing NMDA receptors using novel chicken embryo forebrain cultures and molecular modeling

**DOI:** 10.1002/prp2.480

**Published:** 2019-05-30

**Authors:** Marthe F. Fjelldal, Thibaud Freyd, Linn M. Evenseth, Ingebrigt Sylte, Avi Ring, Ragnhild E. Paulsen

**Affiliations:** ^1^ Department of Pharmaceutical Biosciences School of Pharmacy University of Oslo Oslo Norway; ^2^ Norwegian Defence Research Establishment Kjeller Norway; ^3^ Realomics Strategic Research Initiative Oslo Norway; ^4^ Molecular Pharmacology and Toxicology Department of Medical Biology University of Tromsø—The Arctic University of Norway Tromsø Norway; ^5^ Department of Chemistry Hylleraas Centre for Quantum Molecular Sciences University of Oslo Oslo Norway

**Keywords:** chicken embryo model, EVT‐101, GluN2B antagonists, ifenprodil, in silico modeling

## Abstract

N‐methyl‐d‐aspartate receptors (NMDAR) are widely expressed in the brain. GluN2B subunit‐containing NMDARs has recently attracted significant attention as potential pharmacological targets, with emphasis on the functional properties of allosteric antagonists. We used primary cultures from chicken embryo forebrain (E10), expressing native GluN2B‐containing NMDA receptors as a novel model system. Comparing the inhibition of calcium influx by well‐known GluN2B subunit‐specific allosteric antagonists, the following rank order of potency was found: EVT‐101 (EC_50_ 22 ± 8 nmol/L) > Ro 25‐6981 (EC_50_ 60 ± 30 nmol/L) > ifenprodil (EC_50_ 100 ± 40 nmol/L) > eliprodil (EC_50_ 1300 ± 700 nmol/L), similar to previous observations in rat cortical cultures and cell lines overexpressing chimeric receptors. The less explored Ro 04‐5595 had an EC_50_ of 186 ± 32 nmol/L. Venturing to explain the differences in potency, binding properties were further studied by in silico docking and molecular dynamics simulations using x‐ray crystal structures of GluN1/GluN2B amino terminal domain. We found that Ro 04‐5595 was predicted to bind the recently discovered EVT‐101 binding site, not the ifenprodil‐binding site. The EVT‐101 binding pocket appears to accommodate more structurally different ligands than the ifenprodil‐binding site, and contains residues essential in ligand interactions necessary for calcium influx inhibition. For the ifenprodil site, the less effective antagonist (eliprodil) fails to interact with key residues, while in the EVT‐101 pocket, difference in potency might be explained by differences in ligand‐receptor interaction patterns.

**Abbreviations**
ATD: amino terminal domain
CMV: cytomegalovirus
DMEM: Dulbecco's modified Eagle's medium
FBS: fetal bovine serum
MSV: multiple Sequence Viewer
NMDAR: N‐methyl‐d‐aspartate receptors
PDB: Protein Data Bank
TBS‐T: Tween‐Tris‐buffered saline solution
TGX: Tris‐Glycine‐extended

## 1 INTRODUCTION

The N‐methyl‐d‐aspartate (NMDA) receptors are found in all brain regions and are involved in synaptic plasticity, learning, and memory.^1^ They belong to a subfamily of excitatory glutamate receptors that are ligand‐ and voltage‐gated channels with permeability predominantly for Ca^2+^, but also for Na^+^ and K^+^.^2^ The NMDA receptors consist of heteromeric tetramers built up by the subunits GluN1, GluN2, and GluN3. Two GluN1 and two GluN2 or GluN3 subunits must be present to enable ligand binding. There are four variants of GluN2: GluN2A, GluN2B, GluN2C, and GluN2D, and the structure can be homo‐ or heterotetrameric with respect to the different GluN2 subunits.^3^ The receptor distribution and composition are dynamic and change during development and in response to sensory input.^4^

Neurodegenerative diseases such as Parkinson's disease, Alzheimer's disease, and epilepsy are associated with pathological changes in the assembly and location of NMDA receptors.^5^, ^6^, ^7^ Changes in these receptors are also observed in psychiatric disorders such as schizophrenia and depression.^8^, ^9^ Memantine, a low affinity antagonist that alleviates symptoms of Alzheimer's disease, is one of the very few commercially available drugs targeting NMDA receptors.^10^, ^11^ The severe psychotomimetic side effects observed when administrating unspecific total channel blockers such as MK‐801 and ketamine to humans and animal models,^12^ indicate a need for new partial and/or subtype‐specific antagonists. To develop new drug candidates, it is essential to understand the receptor binding mechanisms and explore the conformational space of the receptor binding sites.

The amino terminal domain (ATD) of the subunits contains binding sites for allosteric compounds, such as the synthetic GluN2B‐specific antagonists eliprodil, Ro 25‐6981, and ifenprodil.^13^, ^14^, ^15^, ^16^ Recently, an x‐ray crystal structure of the GluN1 and GluN2B ATD dimer in complex with ifenprodil was resolved.^17^ However, ifenprodil displays unspecific binding to sigma opioid receptors and monoamine receptors,^18^, ^19^, ^20^ causes behavioral toxicity^21^ and it is readily inactivated by hepatic metabolism.^22^, ^23^ Based on the structural features of ifenprodil, several new GluN2B‐specific antagonists such as Ro 04‐5595, which has been shown to displace Ro 25‐6981, have been investigated.^18^ X‐ray crystal structure complexes showed that EVT‐101 (another GluN2B antagonist) binds at the same GluN1/GluN2B interface as ifenprodil, but occupies an overlapping and less explored binding site.^17^ The GluN2B‐specific allosteric antagonist HON0001 (structurally similar to Ro 04‐5595) has also been shown to have a potent dose‐dependent oral analgesic activity in rats, with less side effects and higher receptor specificity than ifenprodil^24^ and it has been predicted to interact with the EVT‐101 binding site.^17^ In this paper, ligands predicted to bind the ifenprodil‐binding site are denoted A‐ligands, while those predicted to bind the EVT site are named B‐ligands.

The NMDA receptor is evolutionarily conserved across species.^25^ Many studies have therefore used overexpressed chimeric NMDA receptors with constituents from *Rattus norvegicus* (*R. norvegicus*), *Xenopus laevis* (*X. laevis*), or *Homo sapiens* (*H. sapiens*).^26^, ^27^, ^28^ In vitro cultured neurons from the developing chicken brain was recently suggested as a suitable model for nonclinical drug testing.^29^ Chicken forebrain culture expresses native, functional NMDA receptors with a high proportion of GluN2B subunits, features that make them suited for the present study.

In this paper, we utilized chicken embryo primary forebrain culture and a functional calcium influx assay to investigate the potency of GluN2B‐specific allosteric antagonists. Their binding mode was investigated by docking studies and molecular dynamics simulations using experimental structures of GluN1/GluN2B ATD, and the predicted binding data were compared to functional results. We also investigated amino acids critical for ligand binding by in silico mutation studies and found that the residues that differentiate the EVT‐binding site from the ifenprodil site are predicted to be located in the GluN2B subunit. EVT‐101 was significantly more potent than Ro 04‐5595 in terms of calcium influx inhibition, which may be explained by the interaction of EVT‐101 with GluN2BMet134 and GluN2BAla135. When comparing ligands that are predicted to bind to the ifenprodil site, it appeared that the less potent allosteric antagonist eliprodil failed to interact with residues GluN1Ser132, GluN2BTyr175 and GluN2BMet207, all of which display interaction with the stronger inhibitors Ro 25‐6981 and ifenprodil. Among the compounds tested, the ligands proven (ifenprodil and Ro 25‐6981) and predicted (eliprodil) to be located in the ifenprodil‐binding pocket are structurally similar, while the EVT‐101 binding site appears to accommodate more structurally diverse ligands and binding poses, which is supported by earlier work.^17^

## 2 MATERIALS AND METHODS

### 2.1 Chemicals

Dulbecco's modified Eagle's medium (DMEM), penicillin‐streptomycin (Pen‐Strep 100X), N‐2 supplement (100X), GlutaMAX™ supplement, and l‐glutamine were purchased from Gibco™ (part of ThermoFisher Scientific, Waltham, MA). Fetal bovine serum (FBS) and trypsin‐EDTA were acquired from BioWhittaker^®^ (Lonza, Switzerland). Leupeptin, pepstatin A, phenylmethylsulfonyl fluoride, sodium orthovanadate, formaldehyde, and anti‐β‐actin antibody were purchased from Sigma‐Aldrich^©^ (now part of Merck, USA). Triton™ X‐100, Fura‐2 AM cell permeant, and ProLong™ Gold Antifade Mountant with DAPI were obtained from ThermoFisher™ (USA), while anti‐GluN2B came from Abcam (Cambridge, UK). Donkey anti‐rabbit IgG‐HRP was obtained from Santa Cruz Incorporated (Santa Cruz, CA), while Luminata Crescendo and Classico Western HRP substrate and anti‐NeuN antibody came from Merck Millipore (Temecula, CA). FITC A109, anti‐mouse originated from Chemicon International Inc. (later acquired by Merck Millipore), and goat anti‐mouse IgG‐HRP was bought from Biorad (Hercules, CA).

### 2.2 Animals

Fertilized eggs (*Gallus gallus*) from different hatches were purchased from Nortura Samvirkekylling (Våler, Norway). The eggs were incubated at 37.5°C and 45% relative humidity in an OvaEasy 380 Advance EXII Incubator (Brinsea, Weston‐super‐Mare, UK). The viability of the embryos was checked with trans‐illumination using a LED lamp (Brinsea) by observing spontaneous movement. Embryos were sacrificed at embryonic day 10 (E10), and sex determination was not performed. Animals were handled in accordance with the Norwegian Animal Welfare Act and the EU Directive 2010/63/EU. However, chicken embryos are not regarded as research animals before E14 (2010/63/EU; EU, 2010). They have a short incubation time, do not require animal housing and elicit fewer allergies than murine animal models.^30^ It is also easier to predict the number of embryos obtained compared to rat or mice, and the hen is exempted from experiments. Thus, their use is in accordance with the 3Rs principles of animal research.

### 2.3 Chicken embryo forebrain cultures

The eggs were submerged in crushed ice for 7 minutes to anesthetize the embryos before decapitation. The forebrain was surgically removed, and the meninges were discarded. The tissue was homogenized by chopping with a scalpel before trypsination in buffered solutions as previously described.^30^, ^31^ Cells were suspended in DMEM supplemented with 1% N‐2, 100 U/mL penicillin and 0.1 mg/mL streptomycin (Pen‐Strep), 10% fetal bovine serum (FBS), and 0.75% GlutaMAX™. Cells were seeded (1.7 × 10^6^ cells/mL) on 35 mm Petri dishes or in 96‐well plates (Corning^®^ CellBIND^®^ 96 well plates; Merck) precoated with poly‐l‐lysine, and incubated at 37°C, with 5% CO_2_. These cultures contain an abundance of functional GluN2B receptors on DIV1 (A. Ring, pers. commun.).

### 2.4 Transfection of control HEK‐293 cells

Human embryonic kidney cells (HEK‐293 cells, CRL‐1573™ from ATCC^®^, USA) were maintained in DMEM supplemented with 10% FBS, 100 U/mL penicillin and 0.1 mg/mL streptomycin and 4 mmol/L l‐glutamine. Experiments were performed after passage number 3 was reached. The HEK‐293 cells were transfected with K2 Transfection System^®^ (Biontex Laboratories, Munich, Germany), according to the manufacturer's protocol. Briefly, the cells were transfected in 35 mm cell culture dishes at 80% confluency, with 1.42 μg DNA/dish and 4.26 μL K2 solution. The cells were incubated at 37°C and 5% CO_2_ for 24 hours. The transfection efficiency was estimated to be ≥70% by fluorescence microscopy of pEGFP‐N1 (Clontech, USA) transfected HEK‐293 cells. The GluN2A and GluN2B plasmids were kind gifts from Professor S. Vicini (Georgetown University, School of Medicine, Washington, DC), and Dr Luo (Zheijiang University, School of Medicine, China), respectively. An empty vector plasmid containing the cytomegalovirus promoter (CMV plasmid) was a gift from J. Milbrandt (Washington School of Medicine, St. Louis, MO).

### 2.5 Western blotting

Chicken embryo forebrain cultures (harvested at day in vitro 1 [DIV1]) and HEK‐293 cell cultures were washed twice with ice‐cold PBS (4°C) and harvested in 2% SDS (in PBS) added the following protease inhibitors: 5 μg/μL leupeptin, 1 μg/μL pepstatin A, 300 μmol/L phenylmethylsulfonyl fluoride, and 100 μmol/L of the phosphatase inhibitor sodium orthovanadate.

Isolated tissues from chicken forebrain (E7‐18) and mouse cerebellum (postnatal day 21) were frozen in N_2_ (−196°C) before long‐term storage at −20°C. To prepare for western blotting analysis, tissue was homogenized as previously described.^32^ In short, samples were kept on ice, added tris‐EDTA (TE) buffer containing the same protease inhibitors as described above, and homogenized using a motorized pellet pestle. TE with SDS (final concentration 2%) was added to the sample before further homogenization by syringe (25 G) and heat inactivation of proteases (95°C, 5 min). Protein concentration was determined with Pierce™ BCA Protein Assay Kit (ThermoFisher™, USA). Each sample (25 ug) was mixed with Laemmli buffer with 5% mercaptoethanol and then applied to a precast 10‐well polyacrylamide Mini‐Protean Tris‐Glycine‐extended (TGX™) gel (BioRad, Germany). After electrophoresis, the proteins were transferred to a nitrocellulose membrane (TransBlot^®^Turbo™; BioRad, Germany) which was blocked with 5% dry skimmed milk in 1% Tween‐Tris‐buffered saline solution (TBS‐T) for 1 hour at room temperature (RT). The primary GluN2B antibody was diluted in 5% dry skimmed milk in TBS‐T to a concentration of 1:1000 and added to the membranes which were then incubated for 24 hours at 4°C. The membranes were rinsed three times with TBS‐T and incubated for 1 hour at RT with anti‐rabbit secondary antibody (1:10 000 in TBS‐T with 5% dry skimmed milk) before a further rinse cycle with TBS‐T. Bands were detected using chemiluminescence with HRP substrates in the bio‐imaging system Chemi Genius 2 with GeneSnap software (both by Syngene, UK). The amount of internal standard was assessed by immunostaining with β‐actin antibody and anti‐mouse secondary antibody. The data were analyzed using ImageJ software,^33^ and the amount of GluN2B was normalized against the amount of β‐actin protein.

### 2.6 Immunocytochemistry

The cell culture was grown in poly‐l‐lysine‐coated petri dishes with glass bottom (MatTEK Corporation, USA). The cell medium was aspirated. Dishes were added 1 mL of PBS with 3.7% formaldehyde and left at RT for 10 minutes before washing twice with PBS (4°C). The cell membranes were permeabilized with 0.1% Triton‐X in PBS before blocking with 5% dry skimmed milk in 1%TBS/Tween for 30 minutes at RT. After washing twice with cold PBS, the neuronal marker antibody NeuN was diluted in PBS (1:100) and 100 μL was added to the dishes and incubated at 4°C for 12 hours. The dishes were washed three times with cold PBS before 400 μL of secondary FITC antibody diluted in 5% dry skimmed milk in 1%TBS/Tween was added at a concentration of 1:250 and left to incubate in the dark for 1 hour at RT. The cells were mounted with the nuclear marker DAPI and visualized with fluorescence microscopy (Eclipse TE300; Nikon, Japan).

### 2.7 Calcium influx measurement

The procedure was similar to that previously described by Ring et al^34^ Cells were plated in poly‐l‐lysine coated 96‐well black plates with clear glass bottom (Corning^®^ CellBIND^®^) and each well was incubated with 4 μmol/L fluorescent calcium (Ca^2+^) indicator Fura‐2 at 37°C, 5% CO_2_ for 45 minutes.^34^, ^35^ The medium was then replaced with a standard buffer (140 mmol/L NaCl, 3.5 mmol/L KCl, 15 mmol/L Tris (pH 7.4), 1.2 mmol/L Na_2_HPO_4_‐NaH_2_PO_4_ (pH 7.4), 5 mmol/L glucose, and 2 mmol/L CaCl_2_ in distilled water) with 1 mmol/L MgCl_2_ (wash buffer) and further incubated for 15 minutes in the dark for de‐esterification of Fura‐2. Fura‐2 fluorescence was measured using CLARIOstar^®^ plate reader (BMG Labtech, Germany). Intracellular Ca^2+^ changes were expressed as changes in 340/380 nm fluorescence emission ratio. The wash buffer was then carefully replaced with test compound (20 nmol/L to 10 μmol/L) in standard buffer (n = 4 per concentration, two compounds per 96‐well plate). NMDA receptor‐mediated Ca^2+^ influx was induced by NMDA (0.2 mmol/L) and glycine (0.1 mmol/L) in each well. The resulting rise in intracellular Ca^2+^ was expressed as a change in the 340/380 emission ratio by subtracting the initial Ca^2+^ level from the NMDA stimulated Ca^2+^ responses. For the compound EVT‐101, additional experiments with a lower dose range (2 nmol/L to 1 μmol/L) were included due to the low IC_50_ value. Inhibition curves for each compound were established by dose response experiments (n ≥ 5). For Ro 04‐5595, four representative IC_50_ values were chosen to make Figure 2A, while 13 experiments around the median were chosen for Figure 2C.

### 2.8 Sequence analysis and homology modeling

The Schrödinger Suite version 2018‐1 was employed to perform the homology modeling and the docking procedures. Several chimeric x‐ray structures of the ATD domain of the NMDA receptor are available in the Protein Data Bank (PDB).^36^ Among them are *X. laevis*/*H. sapiens* in complex with ifenprodil and EVT‐101 (PDB id: 5EWJ and 5EWM, respectively)^17^, ^37^ and *X. laevis/R. norvegicus* in complex with Ro 25‐6981 (PDB id: 3QEM).^38^ The experimental structures contain two dimers with each dimer consisting of the GluN1 from *X. laevis (*chain A) and the GluN2B from *H. sapiens* or *R. norvegicus* (chain B). Two different conformations of EVT‐101 binding pose can be observed in the crystal structure, depending on what dimer is considered. In this paper, the A dimer was selected for molecular modeling studies. The experimental structures (5EWJ, 5EWM, and 3QEM) were prepared in Protein Preparation Wizard feature in Maestro^39^ by assigning bond orders, adding hydrogen atoms, creating zero‐order bonds to metal and disulfide bonds and building missing loops <20 amino acids (GluN1: amino acid 97‐101, GluN2B: amino acid 53‐62 and 54‐59 for *H*. *sapiens* and *R. norvegicus,* respectively) using Prime.^40^ The large missing loop (186‐209 located in GluN1) was not modeled as it was far from the ligand binding pocket and was therefore not considered to have any impact on the binding pocket. Crystal structure water molecules were retained, and the ionization state of the heteroatoms was handled with a pH of 7.4 ± 0.2. The protonation state of the different residues and the optimization of the hydrogen bonds network were performed with PROPKA at pH = 7.4 ± 0.2 with sampling of the crystal water molecules before a final restrained minimization of heavy atoms.

The chicken GluN1 sequence was retrieved from UniProt (ID: Q4KXT1)^29^ while the chicken GluN2B sequence was retrieved from the predicted target sequence with BLAST (Basic Local Alignment Search Tool, XP_015144845.2, NIH, USA).^30^ The retrieved sequences were aligned with the sequences from chain A and B of the x‐ray crystal structures, using the Multiple Sequence Viewer (MSV) tool. The chicken GluN1 ATD (1‐400 residues) sequence is 91% similar to the GluN1 ATD sequence from *X. laevis*, while the chicken GluN2B ATD has a 95% sequence identity with the human and rat GluN2B ATD. A sequence alignment between human, rat, and chicken Glun2B subunits can be found in supplementary data (Figure S1), made with the Clustal Omega multiple sequence alignment program available at Uniprot's webpage.^41^, ^42^ The total rat and human GluN2B amino acid sequence is 93% similar to the chicken GluN2B sequence, while rat and human GluN2B sequences are 98% similar to each other. The homology model building tool included in MSV was used to construct homology models of each subchain based on the alignment with default settings. Each subunit was merged into a dimer of chicken GluN1 and GluN2B called chicken_NMDA_5EWJ and chicken_NMDA_5EWM, respectively. Finally, the entire model was refined and prepared for docking using the Protein Preparation workflow, which ensured structural accuracy by correcting protein and peptide bond orders, tautomeric and ionization states, and restrained minimization.

The only difference close to the allosteric binding pockets of ifenprodil and EVT‐101 (14.4 and 13.1 Å, respectively), between the chimeric experimental structures and the chicken NMDA receptor, is a valine at position 107 in the chicken GluN1 sequence compared to an isoleucine in position 107 in the *X. laevis* GluN1 sequence. The allosteric binding pocket of the chicken NDMA receptor (chicken_5EWJ and chicken_5EWM) was created by mutating the isoleucine residue in position 107 to valine in the chimeric *X. laevis*/*H. sapiens* and *X. laevis*/*R. norvegicus* NMDA receptor crystal structure (PDB id: 5EWJ and 5EWM respectively). The comparison of the docking poses of the co‐crystallized ligands in the allosteric binding pocket of the chicken vs their binding pose in their respective crystal structure did not reveal any relevant differences (Figure S1A, in supplemental data). Furthermore, the two conformations of EVT‐101 binding pose observed in the crystal structure could be predicted by docking with similar docking scores (Figure S1B). It was therefore decided to use the crystal structures in further docking studies and molecular dynamics simulations.

### 2.9 Ligand preparation and docking studies

The docking procedures were performed in Schrödinger's Glide software.^43^ Receptor grid maps were generated for both crystal structures in complex with ifenprodil and EVT‐101 (PDB id: 5EWJ and 5EWM, respectively) using default settings^44^ and co‐crystallized ligands as the centroid of the map. Two overlapping allosteric binding sites have been described for the GluN1/GluN2B subunits: the ifenprodil and the EVT‐101 binding pockets.^17^, ^38^ In order to study the ligand‐protein interactions of the ligands used in vitro*,* the complexes GluN1/GluN2B: ifenprodil, GluN1/GluN2B: Ro 25‐6981, and GluN1/GluN2B: EVT‐101 were taken from the PDB while GluN1/GluN2B: eliprodil and GluN1/GluN2B: Ro 04‐5595 were generated through docking.

The structure of eliprodil and Ro 04‐5595 was drawn with the software Maestro and prepared using Ligprep (Schrödinger Release 2018‐1: LigPrep, Schrödinger, LLC, New York, NY, 2018). Enantiomers and protonation states at a target pH = 7.4 ± 0.2 were generated. Stroebel et al^17^ reported that the GluN1 residues leucine 135 and isoleucine 133 rotate to fill the empty space of the ifenprodil‐binding pocket when EVT‐101 is co‐crystallized with the NMDA receptor, obstructing the binding pocket of ifenprodil. Hence, eliprodil and Ro 04‐5595 were docked into both chimeric *X. laevis*/*H. sapiens* NMDA x‐ray crystal structures using the virtual screening workflow. Standard precision was employed with retention of three docking poses per enantiomer for a final MM‐GBSA calculation. For each ligand, the complex protein‐docking pose with the best MM‐GBSA score was chosen as input for the MD simulation.

Due to its structural similarity with Ro 04‐5595 and interesting pharmacological properties, HON0001 was compared to Ro 04‐5595 using the MOLPRINT2D fingerprint and Tanimoto similarity metrics in the Canvas software (Schrödinger Release 2017‐3: Canvas, Schrödinger, LLC, New York, NY, 2017), obtaining a Tanimoto score (similarity) of 0.750.

### 2.10 Molecular dynamics simulations

Molecular dynamics simulations were performed with the Desmond program.^45^ The selected complexes were set up in an orthorhombic simulation box with periodic boundary condition, the OPLS3 force field TIP3 water model was employed for the solvation of the system before it was neutralized and 0.15 mol/L NaCl was added. The generated systems were relaxed using the Desmond default protocol and run for 100 ns on a GPU. The NPT ensemble was selected with a *P* = 1.01325 bar and T = 300 K using the Martynas‐Tobias‐Klein barostat method (relaxation time of 2 ps and isotropic coupling style) and the Nose‐Hoover Chain thermostat method (relaxation time of 1 ps and one group for temperature), respectively. The RESPA integrator was selected and the bonded and close nonbonded interactions were handled with a timestep of 2 fs while for far nonbonded interactions the timestep was set to 6 fs. A cut‐off of 9 Å was used for the short‐range columbic interactions. The trajectories and energies were recorded every 10 ps giving a total of 10 000 frames. Root Mean Square Deviations of protein and ligand can be observed in Figure S3. The last 10 ns (90‐100 ns corresponding to the last 1000 frames) were considered for analysis of the protein–ligand interactions, the generation of average ligand–receptor complexes, and alanine scanning calculation utilizing the residue scanning tool from BioLuminate.^46^

All residues within 5 Å of the ligand in the averaged complexes were mutated into alanine and their contribution to the free energy of binding (ΔG) was analyzed by calculating the difference in ΔG before and after mutation for each residue. The averaged conformation of eliprodil and EVT‐101's receptor‐ligand complex required additional minimization before alanine mutation scanning could be performed. This was done by the minimization panel featured in the MacroModel software,^47^ with OPLS3 force field, water as solvent and extended cut‐off.

### 2.11 Analysis and statistics

Outlier values were tested for by the built‐in feature in GraphPad (Robust regression and Outlier removal, *Q* = 1%) and normality was checked with the D'Agostino‐Pearson omnibus normality test. Statistically significant differences were evaluated by Kruskal‐Wallis’ test or Mann‐Whitney's test depending on the number of samples. Dunn's multiple comparison test was included as post hoc*‐*test when appropriate.

## 3 RESULTS

### 3.1 Cultures from chicken forebrain express GluN2B

**Figure 1:**
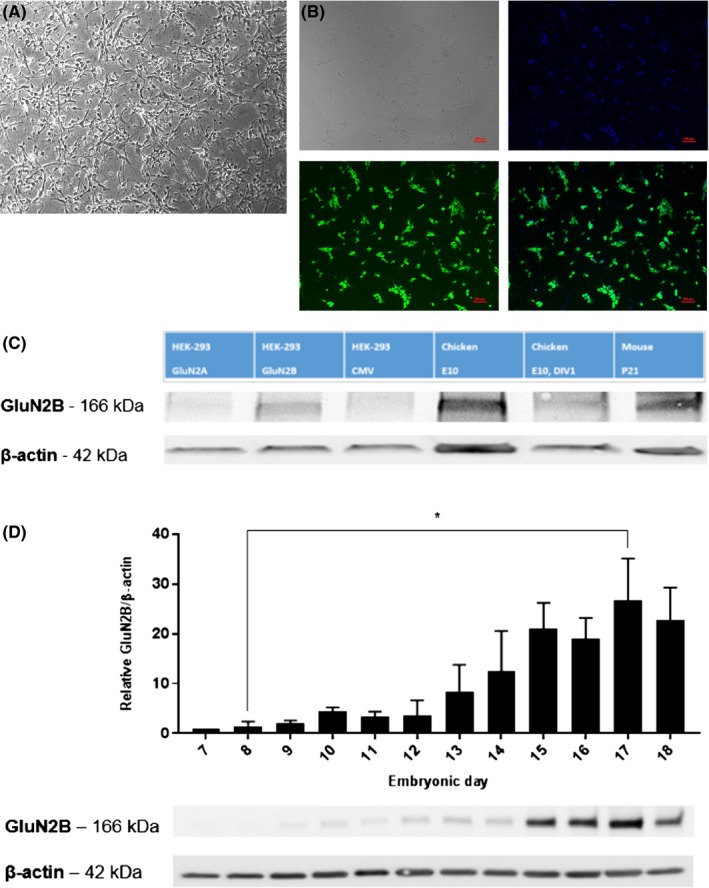
Embryonic chicken forebrain neurons can be grown in vitro and GluN2B is expressed in chicken forebrain tissue in the fetal period. Chicken forebrain was harvested at E10 and the cell culture was incubated overnight, before inspected at DIV1, using: A, Light microscopy and B, Immunostaining with neuronal marker NeuN and nuclear stain DAPI. B1: Brightfield image. B2: Staining with DNA marker DAPI, visualized by UV light. B3: Immunostaining with NeuN, visualized by fluorescence microscopy. B4: Composite image of DAPI stain and NeuN immunostaining. C: Western blot stained with anti‐GluN2B antibody, concentration 1:1000 (ab65783, Abcam, UK) and anti‐β‐actin antibody. Lanes: 1‐3: HEK cells transfected with GluN2A, GluN2B or CMV plasmid, respectively. Lane 4: Homogenized chicken forebrain tissue harvested at E10. Lane 5: Chicken embryo forebrain cell culture, harvested at E10 and analyzed at DIV1. Lane 6: Homogenized mouse cerebellum harvested at P21. D: Time series of GluN2B protein expression in homogenized chicken embryo forebrains, from E7 to E18. GluN2B protein expression relative to internal control protein β‐actin expression. The values are normalized to expression level at E7 (n = 3), and statistical significance was investigated using the Kruskal‐Wallis test. Variation is given as standard deviation and a representative example of western blot of GluN2B and β‐actin is shown below the graph

Since chicken primary forebrain neuron cultures have not been described before, we immunostained them with NeuN, a marker of most neurons that have reached a certain level of maturity. The fraction of NeuN‐positive cells was estimated to be 40% relative to the overall cell number (DAPI‐stained nuclei) at DIV1 (Figure 1A,B).

The presence of GluN2B was confirmed with western blotting. Specificity of the GluN2B antibody was assessed in transfected HEK‐293 cells overexpressing the GluN2B subunit, shown as a strong band at 166 kDa (consistent with the expected M_w_ of GluN2B) (Figure 1C). No band in this range was detected in the negative control samples (HEK‐293 cells transfected with control vector CMV or GluN2A subunit plasmid). Both chicken embryo forebrain tissue (E7‐18), mouse cerebellar tissue (postnatal day [P] 21), and cultures from chicken forebrain (DIV1) expressed GluN2B. The relative level of GluN2B protein in the chicken forebrain increased rapidly from E12 and reached a plateau at E15 (Figure 1D).

### 3.2 Functional properties of chicken NMDA receptors resemble their human and rat counterparts

**Figure 2:**
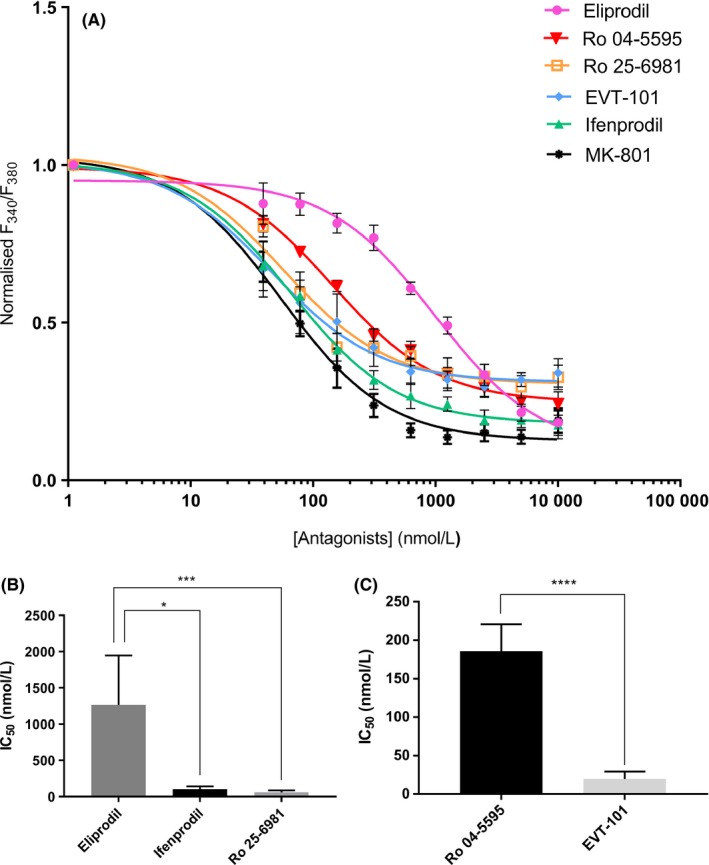
Inhibition curves and statistical comparisons of IC_50_‐values of A‐ligands and B‐ligands. IC_50_ values in chicken were determined by calcium influx measurement in E10 chicken forebrain cell culture at DIV 1. Compounds were tested with twofold dilution series from 10 μmol/L to 20 nmol/L. Calcium influx was induced with NMDA and glycine (200 and 100 μmol/L concentration, respectively) and the intracellular calcium level was measured by the fluorescent ratiometric Fura‐2 assay as described in Material and Methods. A, Normalized inhibition curves for EVT‐101, Ro 25‐6981, MK‐801, ifenprodil, Ro 04‐5595 and eliprodil. Variation is presented as SEM (n = 4‐8). B, Comparison of IC_50_‐values of A‐ligands predicted to bind the ifenprodil‐binding pocket. Statistically significant differences were established with Kruskal‐Wallis’ test and Dunn's multiple comparison test which gave a statistically significant difference of ** (adjusted *P*‐value ≤ 0.01) between eliprodil and Ro 25‐6981 (n = 5‐8). C, Comparison of IC_50_ values of B‐ligands predicted to interact with the EVT‐101 binding pocket. Differences were investigated with Mann‐Whitney test giving a statistically significant difference of **** (*P*‐value = 0.0001) between EVT‐101 and Ro 04‐5595 (n = 8‐13). For B and C, variation is presented as SD

Functional properties of the NMDA receptor in chicken forebrain culture were tested with the calcium influx assay as described previously.^34^ It was shown that the receptor was activated by standard protocol concentrations of NMDA and glycine. The NMDA receptors in the cultures were assumed to contain a significant fraction of GluN2B subunits as approximately 70% of the calcium influx could be blocked by the GluN2B‐specific allosteric antagonists Ro 25‐6981, ifenprodil, eliprodil, and EVT‐101, at concentrations shown to elicit similar responses in rat and human NMDA receptors (Table 1 and Figure 2A)^17^, ^48^ and that the calcium influx was reduced by 90% by 10 μmol/L of the unspecific NMDA receptor inhibitor MK‐801.^49^, ^50^ The less‐explored antagonist Ro 04‐5595 showed an IC_50_ value of <200 nmol/L. The differences in IC_50_ values between eliprodil and the other A‐ligands were statistically significant: Eliprodil vs ifenprodil and eliprodil vs Ro 25‐6981 (*P ≤ 0.05, and ***P ≤ 0.001, respectively, shown in Figure 2B). B‐ligands EVT‐101 and Ro 04‐5595 gave significantly different IC_50_ values when tested experimentally in the chicken forebrain primary culture calcium influx assay (****P < 0.0001, Figure 2C).

**Table 1:** IC_50_ values of GluN2B‐specific allosteric antagonists across species

Compound	Chicken (present work)		Human^48^		Rat^17^, ^48^	
	IC_50_	n	IC_50_	n	IC_50_	n
Ro 25‐6981	60 ± 27	6	49 ± 8	8	42 ± 6	4
Ifenprodil	103 ± 39	5	130 ± 10	6	110 ± 10	5
Eliprodil	1263 ± 683	8	930 ± 140	4	780 ± 90	6
EVT‐101	22 ± 8	8	–	–	12 ± 0.2	12
Ro 04‐5595	186 ± 35	13	–	–	–	–

IC_50_ values for Ro 25‐6981, ifenprodil, eliprodil, EVT‐101, and Ro 04‐5595. IC_50_ values in chicken neurons were determined by calcium influx measurement in E10 chicken forebrain cell culture at DIV 1. Compounds were tested with twofold dilution series from 10 μmol/L to 20 nmol/L. Calcium influx was induced with NMDA and glycine (200 and 100 μmol/L concentration, respectively) and the intracellular calcium level was measured by the fluorescent ratiometric Fura‐2 assay as described in Material and Methods. Published data from human and rat were determined by electrophysiology in recombinant GluN1/GluN2B receptors expressed in *X. laevis* oocytes (data from 48 and 17).

### 3.3 Computer modeling reveals conserved tertiary structure of chicken GluN1/GluN2B ATD

The high percentage of amino acid sequence similarity between chicken GluN1/GluN2B ATD and *X. laevis/H. sapiens* GluN1/GluN2B ATD provided a good starting point for making a homology model of the chicken GluN1/GluN2B ATD (Figure S1). Introducing an in silico mutation of the only divergent amino acid close to the ifenprodil/EVT‐101 binding site in the crystal structures into the corresponding amino acid in the chicken, GluN1Ile107 to GluN1Val107 in chicken, did not affect the binding poses and properties of ifenprodil or EVT‐101 compared with the x‐ray complexes (supplemental data, Figure S2). These similarities enabled the use of experimental x‐ray structures instead of the chicken homology model for studying the dynamics of ligand interactions. X‐ray structures are regarded as both structurally and energetically more stable than homology models and more reliable predictions are expected.

### 3.4 Molecular dynamics simulations predict interactions between ligands and binding site residues

**Figure 3:**
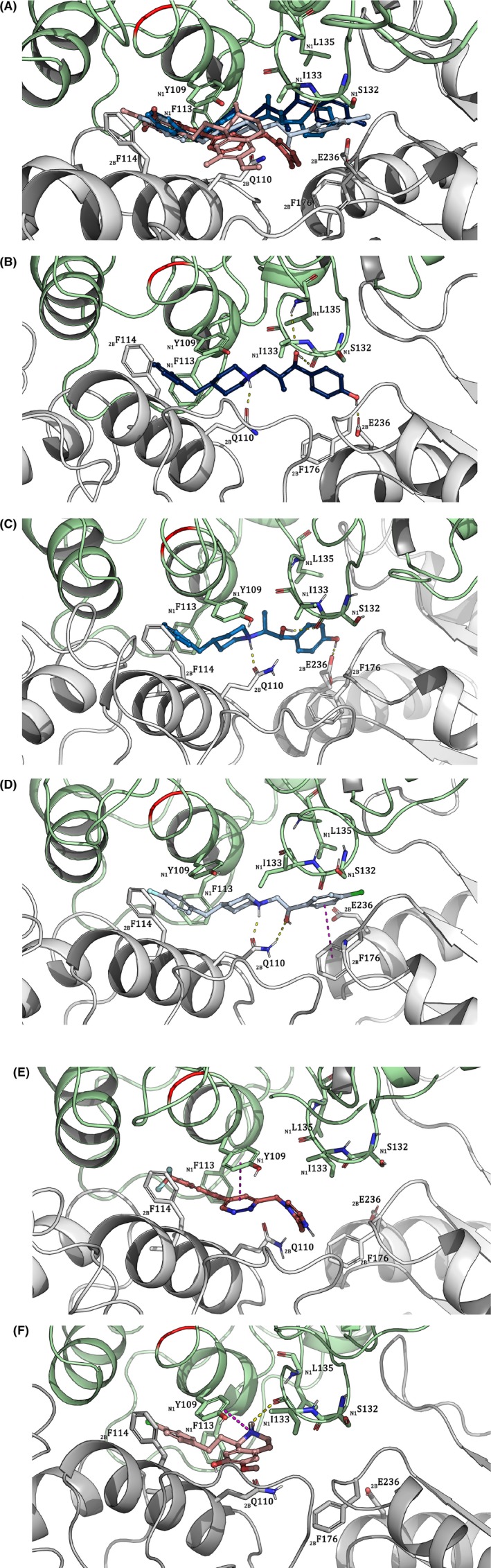
Representation of the average binding mode of each ligand with display of important binding site residues in their respective average protein structure. The bright red band indicates the position of the divergent residue in the chicken NMDA receptor. N1 and 2B prefix denote GluN1 and GluN2B, respectively. Yellow dashes represent hydrogen bonds and magenta dashes π‐stacking/π‐cations. A‐ligands are blue, B‐ligands are red. A, Overview of the binding poses of Ro 25‐6981, ifenprodil, eliprodil, Ro 04‐5595, and EVT‐101, combined. B, Ro 25‐6981 C, Ifenprodil, D, Eliprodil, E, EVT‐101 F, Ro 04‐5595

Our docking studies and molecular dynamic simulations supported that Ro 25‐6981 and ifenprodil shared the ifenprodil‐binding site and showed that eliprodil interacted with the ATD domain via the ifenprodil‐binding site. This is supported by earlier findings.^17^, ^38^, ^51^ Eliprodil gave a Molecular Mechanics/Generalized Born Surface Area (MM‐GBSA) score of −93.65 kcal/mol when docked in the ifenprodil‐binding pocket vs a −70.75 kcal/mol MM‐GBSA score when docked in the EVT‐101 binding pocket. The calculations also predicted that EVT‐101 and Ro 04‐5595 bound to the less explored EVT‐101 binding site, sharing a hydrophobic pocket with the ifenprodil‐binding site.^17^ The best MM‐GBSA score for Ro 04‐5595 was −71.23 kcal/mol in the EVT‐101 binding pocket vs −61.84 kcal/mol in the ifenprodil pocket. An overview of the overlapping binding poses and residue interactions of Ro 25‐6981, ifenprodil, eliprodil, EVT‐101, and Ro 04‐5595 are shown in Figure 3 and Table 2, respectively. Individual binding poses and selected interactions are shown in Figure 3B‐F.

**Table 2:** Overview of predicted binding residues

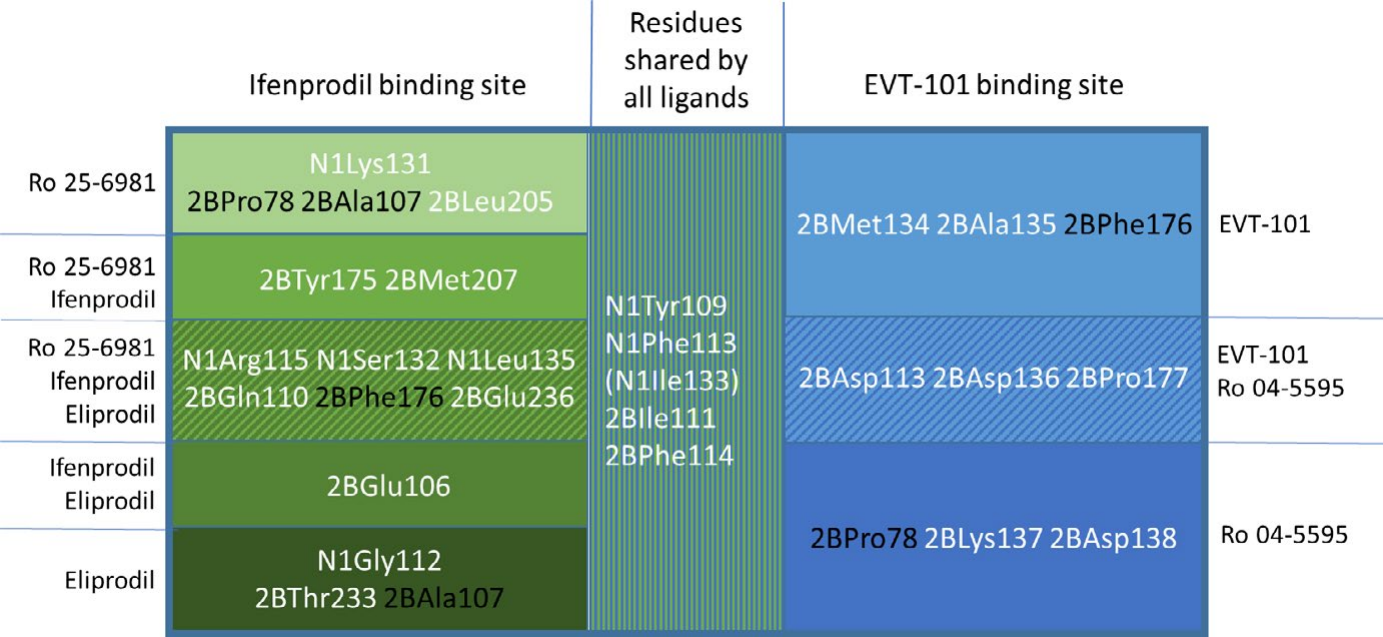

A schematic overview of the shared binding residues, binding residues in each binding pocket and specified to ligands within each binding pocket. Prefix N1 denotes that the residue is located in the GluN1 subunit, while 2B indicates the GluN2B subunit. Residues shared between ligands are shown in black: 2BPro78 is shared between Ro 25‐6981 and EVT‐101, 2BPhe176 is common for ifenprodil, eliprodil, and EVT‐101, while 2BAla107 is predicted to bind both Ro 25‐6981 and eliprodil.

#### 3.4.1 The common hydrophobic pocket

**Figure 4:**
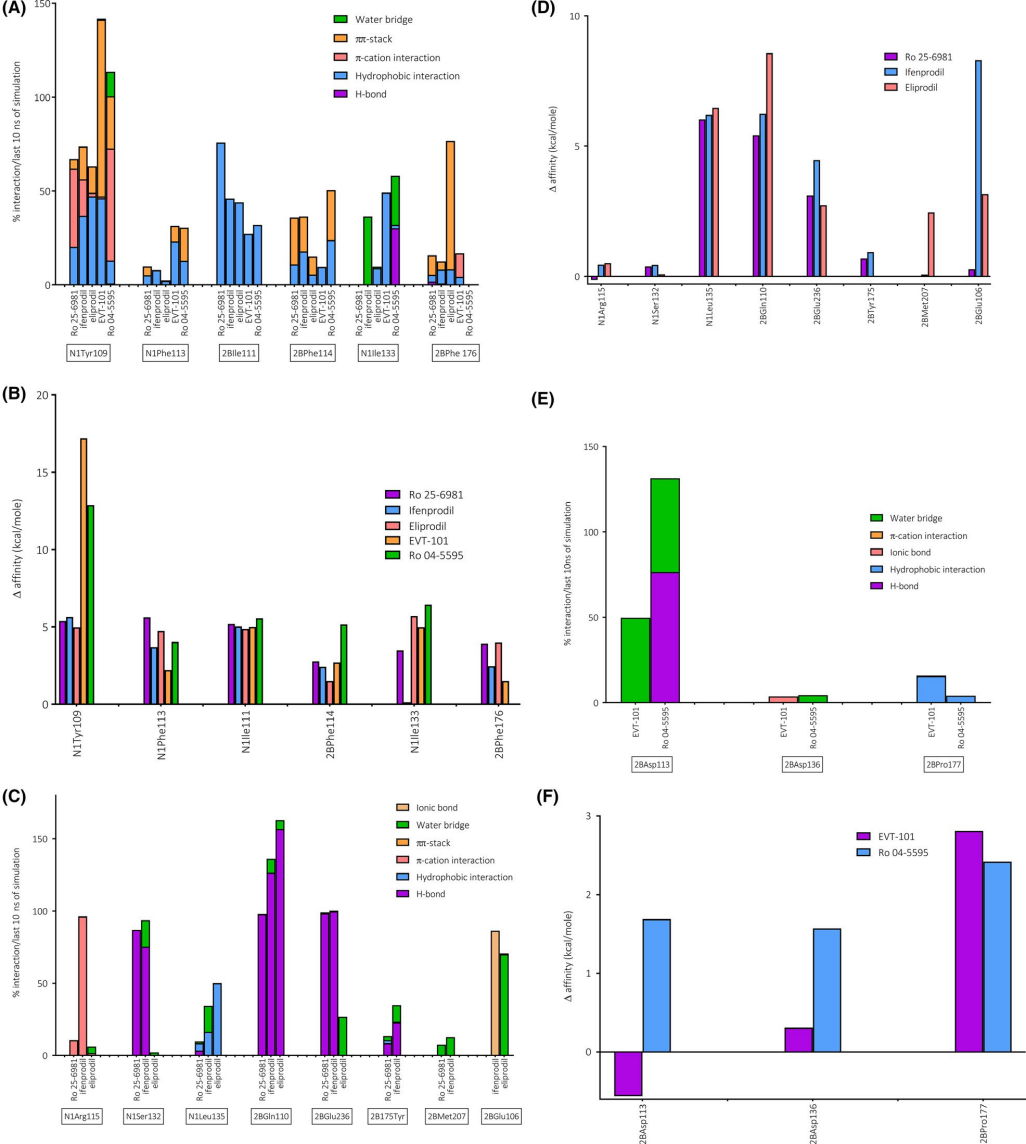
Observed interactions between the ligands and the receptor during molecular dynamics simulations and comparison of the free energy of binding ΔG (kcal/mole) before and after alanine mutation scanning. The percentage of frames (complexes) showing interactions between the ligands and their binding amino acids during the last 10 ns of the molecular dynamics simulations (1000 frames). The interactions comprise H‐bonds, π‐cation interaction, π‐π stacking, other hydrophobic interactions, ionic bonds, and water bridges. One residue can have several interactions, which is why some values exceeded 100%. Prefix N1 denotes that the residue is located in the GluN1 subunit, while 2B indicates the GluN2B subunit. A, The interactions between Ro 25‐6981, ifenprodil, eliprodil, EVT‐101, Ro 04‐5595, and the residues shared by all ligands: N1Tyr109, N1Phe113, N2BIle111, and N2BPhe114. N1Ile133 was shared by all except Ro 25‐6981 and 2BPhe176 was shared by all except Ro 04‐5595. B, Differences in the free energy of binding (ΔG) (kcal/mole) for Ro 25‐6981, ifenprodil, eliprodil, EVT‐101 and Ro 04‐5595 when mutating residues shown in 4A into alanine. C, The interactions between Ro 25‐6981, ifenprodil, eliprodil, and their shared residues located in the ifenprodil‐binding site: N1Arg115, N1Ser132, N1Leu135, 2BGLN110 and 2BGlu236. D, Δ affinity (kcal/mole) for Ro 25‐6981, ifenprodil and eliprodil when mutating residues shown in 4C into alanine. E, The interactions between EVT‐101 and Ro 04‐5595 and their shared residues located in the EVT‐101‐binding site: 2BAsp113, 2BAsp136, and 2BPro177.F, Δ affinity (kcal/mole) for EVT‐101 and Ro 04‐5595 when mutating residues shown in 4E into alanine

Docking indicated that a part of the binding pocket is common for all compounds. This region of the receptor is hydrophobic and includes Tyr109 and Phe113 in GluN1, as well as Ile111 and Phe114 in GluN2B which all accommodate an aromatic ring, or the interface toward the linker region of the ligands.^51^ In addition, all ligands were predicted to interact with GluN1Ile133 (Ro 25‐6981 interacted sporadically), and all except Ro 04‐5595 were predicted to interact with GluN2BPhe176. Molecular dynamics simulations suggested that all ligands formed nontransient interactions with GluN1Tyr109, but B‐ligands were predicted to have the most stable interaction with this residue (Figure 4A). Mutating Tyr109 into alanine and calculating the change in binding free energy did indeed predict a more substantial drop in affinities for B‐ligands than for A‐ligands. The B‐ligands were also predicted to interact more strongly with GluN1Phe113. Despite that, alanine mutation scan of GluN1Phe113 predicted quite similar changes in affinity for Ro 25‐6981, eliprodil and Ro 04‐5595, while ifenprodil and EVT‐101 had a lesser decrease in affinity compared to the other ligands. GluN2BIle111 was predicted to have quite similar interaction with all ligands, reflected by the uniform effect of the alanine mutation on ΔG values. Of all, Ro 04‐5595 was predicted to have the most stable interaction with GluN2BPhe114, followed by ifenprodil and Ro 25‐6981. This was supported by alanine mutation scanning data, predicting the largest change in ΔG for Ro 04‐5995 when mutating GluN2BPhe114 into alanine in silico.

Ifenprodil, EVT‐101, and Ro 04‐5595 were predicted to bind GluN1Ile133 equally firmly, but with different bonding patterns. Ifenprodil interacted with GluN1Ile133 through a water bridge (Figure 4A), while eliprodil displayed less stable interaction than the others with GluN1Ile133. However, all ligands except Ro 25‐6981 received a comparable reduction in affinity when GluN1Ile133 was mutated into alanine. GluN2BPhe176 on the other hand was predicted to interact with Ro 25‐6981, ifenprodil and EVT‐101 in a fairly similar manner, while eliprodil displayed a very stable interaction to the residue. Despite that, the affinities of Ro 25‐6981 and eliprodil were lowered similarly by alanine mutation, while the affinities of ifenprodil and EVT‐101 were affected to a lesser degree. The affinity of Ro 04‐5595 was not affected, as expected (Figure 4B).

#### 3.4.2 The ifenprodil‐binding site

The predicted common residues for the A‐ligands Ro 25‐6981, ifenprodil and eliprodil were Arg115, Leu135, Ser132 (GluN1), Gln110, and Glu236 (GluN2B). Mutating GluN1Arg115 did not noticeably affect the affinity for any of the ligands. For ifenprodil, this was quite surprising, as it was predicted to have a stable interaction with the residue. This may be explained by the distance between ifenprodil and the positive charge of the residue, which exceeds 5 Å. Both Ro 25‐6981 and ifenprodil were predicted to bind firmly to GluN1Ser132, while eliprodil was only predicted to be loosely associated with this residue (Figure 4C). Alanine scanning mutation showed minimal difference in affinity for all three ligands, as they interact primarily with the amino acid backbone. GluN1Leu135 displayed the largest amount of interaction with eliprodil and slightly less with ifenprodil, while Ro 25‐6981 was predicted to bind GluN1Leu135 to a lesser extent. However, the predicted affinities were similarly affected for all ligands when mutating the residue into alanine. Molecular dynamics simulations predicted comparable interaction properties for all ligands toward GluN2BGlu110. Alanine mutation scanning supported this result giving quite similar ΔG values, but with the largest decrease in affinity for eliprodil. Eliprodil was anticipated to interact the least with GluN2BGln236, while both Ro 25‐6981 and ifenprodil displayed stable bonding patterns. Despite that, affinities of Ro 25‐6981 and eliprodil were lowered quite equally when mutating the residue in silico, though the affinity of ifenprodil was more affected.

Both Ro 25‐6981 and ifenprodil were predicted to bind GluN2BTyr175 and GluN2BMet207, although ifenprodil displayed a more stable interaction. Both ligands were predicted to have comparable loss of affinity when mutating GluN2BTyr175 into alanine, while mutating GluN2BMet207 into alanine only slightly decreased the affinity of ifenprodil. Eliprodil and ifenprodil were predicted to both have a stable binding to GluN2BGlu106. Affinity of ifenprodil to the binding site was predicted to be severely decreased by mutating the residue to alanine, while the affinity of eliprodil was less affected, suggested to be caused by its bond type (π‐π stack vs water bridge). According to the molecular dynamics simulations Ro 25‐6981 interacted with two amino acid residues on its own: GluN1Leu131 and GluN2BLeu205. Both were weak hydrogen bond interactions. Mutation of both residues into alanine was not predicted to have an extensive effect on the affinity of Ro 25‐6981 to the binding site (Figure 4D).

#### 3.4.3 The EVT‐101 binding site

The amino acid residues Asp113, Asp136, and Pro177 (GluN2B) were predicted to interact with both B‐ligands Ro 04‐5595 and EVT‐101. GluN2BAsp113 presumably displays a stable interaction to Ro 04‐5595 and a weaker connection to EVT‐101. Introducing in silico mutations of asparagine to alanine had an opposite effect on the affinity of the two ligands. The affinity of Ro 04‐5595 was lowered, while EVT‐101 was predicted to bind the mutant stronger. Both predicted interactions with GluN2BAsp136 appeared in <5% of the last 10 ns of simulation time, reflected by the relatively low impact on affinity when mutating the residue into alanine. GluN2BPro177 was predicted to have interactions with both EVT‐101 and Ro 04‐5595, and the effect of changing the residue to alanine was relatively similar for both ligands. EVT‐101 appears to have a hydrophobic interaction with GluN2BMet134 and a stable H‐bond with the backbone of GluN2BAla135 and the effect on the affinity of the ligand when mutating the methionine residue was noticeable. Ro 04‐5595 was predicted to interact weakly with GluN2BLys137 and GluN2BAsp138, mainly through water bridges. Alanine mutation scanning indicated an increase in ligand affinity when mutating both residues to alanine. The residues that were predicted to interact with both EVT‐101 and Ro 04‐5595 are shown in Figure 4E and the corresponding alanine mutation scanning results are shown in Figure 4F.

Some of the residues that did not interact with the ligands still affected their affinities when running an alanine mutation scan, probably due to local conformational changes within the binding cavities or indirect effects. The residues are summarized in Figure S4 in supplementary data, and we found that Ro 25‐6981, ifenprodil, and eliprodil shared some of them. The only residue shared by all ligands is GluN1Thr110, for which an alanine mutation is predicted to be especially critical for the affinity of EVT‐101, but enhances the affinity of Ro 04‐5595. Overviews of all predicted interactions and the ΔG differences (kcal/mole) for all residues and ligands predicted by alanine mutation scanning are included in the supplementary data (Figure S5).

## 4 DISCUSSION

In the present study, we have used primary cultures from chicken embryo forebrain as a model to study potencies of different GluN2B polyamine site antagonists to reduce calcium influx. To support the experimental data, computational methods were applied to predict binding to amino acids in the two overlapping ifenprodil and EVT‐101 sites.

The chicken embryo forebrain cell culture expresses GluN2B, established by using a specific antibody raised against a rat GluN2B epitope. Compared to human and rat, the expression pattern of GluN2B in developing chicken forebrain follows a similar trajectory. However, the decline in GluN2B protein expression appears to take place prenatally in chicken, as opposed to postnatally in human and rat. This may reflect a higher degree of relative maturity of the cortex in newly hatched chickens compared to new‐born rats or humans. This is an advantage when considering chicken embryos as an animal model for NMDA receptor development, as it enables easy access to study developmental processes occurring postnatally in other research animals, while the chicken is still contained within the egg.

Since expression studies confirmed the presence of GluN2B‐containing receptors, we wanted to confirm that these were functional in vitro. This was done by studying NMDA‐ and glycine‐induced calcium influx. However, it is important to note that the chicken forebrain cell culture contains different cell types, with approximately 40% mature neurons (NeuN positive). As the NMDA‐induced Ca^2+^ influx could be inhibited 70% by GluN2B‐specific antagonists and 90% with the nonspecific NMDA receptor blocker MK‐801, we assume that some of the Ca^2+^ influx originated from NMDA channels with a different subunit composition. Naturally, this contributes to the larger standard deviations we observed in our experiments, compared to that observed in pure, transfected GluN1/GluN2B receptors. Still, chicken E10 embryo primary forebrain cultures proved to be an effective and reproducible way of accessing native, functional GluN2B‐containing NMDA receptors. The difference in the chicken CTD compared to human (88% similarity) may reduce the validity of the chicken model in experiments regarding downstream NMDA receptor signaling, but as we obtained similar IC_50_ values for known GluN2B allosteric antagonists in chicken that have previously been described for rat and human^48^, ^52^ it is most likely that the human, rat, and chicken receptors share similar functional properties.

The significant differences in IC_50_ values between the antagonists tested suggest different binding properties and these were investigated in silico by docking studies and molecular dynamics simulations, predicting temporal information on the interactions between ligands and binding residues as well as providing details on bond types. As no experimental structures of NMDA receptor in complex with eliprodil and Ro 04‐5595 were available at the time of the writing, the molecular modeling approach gave new knowledge about the binding properties of these compounds. The only nonidentical amino acid (GluN1Val107 in the chicken, vs Glun1Ile107 in the *X. laevis/H. sapiens* crystal structure) close to both binding sites in the chicken homology model did not have any effect on the docking pose of the antagonists ifenprodil and EVT‐101 compared to that of the x‐ray structures, because valine and isoleucine rotamers were predicted to point away from the binding pockets, which implies less probability of influence on the binding. None of the ligands were predicted to interact with GluN1Ile107 so the residue does not appear to be important for either the ifenprodil or EVT‐101 binding pocket.

Of the three ligands that bind the ifenprodil‐binding site, Ro 25‐6981 and ifenprodil were the most effective GluN2B subunit‐specific Ca^2+^ influx inhibitors when tested in the chicken forebrain primary cell culture assay, supported by the work of Hedegaard et al,^48^ in rat. Our in vitro experiments showed a significant difference in IC_50_ value between Ro 25‐6981 and ifenprodil vs eliprodil, which was thus addressed in the in silico studies with supporting findings: Eliprodil was predicted to interact less with GluN1Phe113, GluN1Ser132, GluN2BPhe114, GluN2BMet207 and GluN2BGlu236, but more with GluN2BPhe176 than the rest of the ligands. Of these, GluN1Ser132, GluN2BMet207, and GluN2BGlu236 have been cited as important binding residues for known GluN2B‐specific allosteric inhibitors binding the ifenprodil‐binding pocket in earlier publications.^13^, ^17^, ^38^, ^51^ Mutel et al^18^ found Ro 25‐6981 to have larger affinity to the binding site than both ifenprodil and eliprodil and it might be suggested that the predicted interactions with GluN1Lys131, GluN2BPro78 and GluN2BLeu205 granted the Ro 25‐6981 a better ability to inhibit Ca^2+^ influx than the other A‐ligands. Computational mutation of these residues showed that of these, only mutation of GluN2BPro78 into alanine was predicted to have larger effect on the affinity of Ro 25‐6981 than the other A‐ligands. However, the weak bonds with GluN1Lys131 and GluN2BLeu205 may still be involved in the antagonistic effect of the ligand.

In silico docking of the less‐explored GluN2B‐specific allosteric antagonist Ro 04‐5595 predicted that it bound to the recently discovered EVT‐101 binding pocket. This conclusion is supported by docking studies of the structurally similar compound HON0001^24^ which predicted that HON0001 would bind to the EVT‐101 site as well.^17^ The analgesic effect of orally administrated HON0001 encourages further investigations of Ro 04‐5595 as a potential research tool or drug.

The IC_50_‐values of EVT‐101 and Ro 04‐5595 were significantly different, with EVT‐101 as the most effective antagonist. Compared to EVT‐101, Ro 04‐5595 was predicted to interact more strongly with GluN2BPhe114 and much less with GluN2BPhe176, and this is supported by the alanine scanning results. It appears that EVT‐101 and Ro 04‐5595 are predicted to interact with *different* residues to a larger degree than in the ifenprodil site, rather than Ro 04‐5595 failing to interact with important residues, as might be the case with eliprodil. This is supported by the work of Stroebel et al^17^ who predicted more diverse binding poses of the ligands docked in the EVT‐101 binding pocket, compared to the ligands docked in the ifenprodil‐binding pocket. EVT‐101 was predicted to interact with GluN2BMet134, GluN2BAla135 and GluN2BPhe176 (shared with the A‐ligands) on its own, and has a much stronger interaction with GluN2BPro177 than Ro 04‐5595. Of these, in silico alanine mutation of GluN2BMet134 decreased the ligand affinity noticeably. Ro 04‐5595 supposedly interacts with GluN1Leu135 (shared with all the A‐ligands), GluN2BPro78 (shared with Ro 25‐6981), GluN2BLys137 and GluN2BAsp138 alone, where the interaction with the last two mentioned may be less favorable in terms of causing an inhibiting effect on Ca^2+^influx. All of these interactions had corresponding alanine mutation scanning results. Earlier mutagenesis experiments changing GluN2BAla135 to proline, GluN2BPhe176 to alanine, and GluN2BPro177 to cysteine did indeed increase the IC_50_ value of EVT‐101.^17^

Stroebel et al^17^ have analyzed three in vitro alanine mutations. They observed that in vitro mutation of GluN1Ile133 led to a lower IC_50_ value for ifenprodil, and a higher value for EVT‐101. This corresponded with our in silico observation of higher loss of affinity for EVT‐101 than for ifenprodil. However, mutation of GluN1Leu135 to alanine in vitro*,* which gave small changes in IC_50_ values, did not correspond with our in silico alanine mutation scan results, which predicted a reduction in both ifenprodil and EVT‐101 affinities. Also, in vitro alanine mutation of GluN2BPhe176 increased the IC_50_ values of both ligands drastically. Interestingly, only the least efficient antagonist, eliprodil, was predicted to have a stable interaction with this residue, and ifenprodil and EVT‐101 affinities were predicted to be less affected by this mutation. These discrepancies underscore the importance of comparing in silico data with experimental data.

In conclusion, we have established the chicken primary forebrain culture as a useful, reliable and convenient model to study functional properties of native GluN2B‐containing NMDA receptors, giving experimental support to in silico binding studies. The less investigated GluN2B‐specific allosteric antagonist Ro 04‐5595 was predicted to interact with the novel EVT‐101 binding site, an interesting pharmaceutical target as it mediates a high degree of calcium influx inhibition when bound. The EVT‐101 binding pocket accommodates more structurally diverse ligands compared to the well‐known ifenprodil site and contains interesting binding residues such as GluN2BMet134 and GluN2BAla135. Eliprodil was predicted to interact less with the ifenprodil‐binding site than Ro 25‐6981 and ifenprodil, supporting our in vitro experiments where it presented as the least potent antagonist.

## DISCLOSURES

None declared.
