# Supplementary material for: Exploring the overlapping binding sites of ifenprodil and EVT‐101 in GluN2B‐containing NMDA receptors using novel chicken embryo forebrain cultures and molecular modeling
Source: Pharmacol Res Perspect. 2019 May 30;7(3):e00480. doi: 10.1002/prp2.480 (PMC6543015; doi:10.1002/prp2.480)
Supplement: Supplementary file 1 [file PRP2-7-e00480-s001.pdf]

Supplemental Data

Pharmacology Research & Perspectives

Exploring the overlapping binding sites of ifenprodil and EVT-101 in GluN2B containing NMDA receptors using novel chicken embryo forebrain cultures and molecular modelling

M. F. Fjelldal[1,2,3], T. Freyd [4], L. M. Evenseth[4], I. Sylte[4], A. Ring[2], R. E. Paulsen[1,3].

[1] Department of Pharmaceutical Biosciences, School of Pharmacy, University of Oslo, Oslo, Norway, [2]Norwegian Defence Research Establishment, Kjeller, Norway, [3]Realomics Strategic Research Initiative, Oslo, Norway, [4]Molecular Pharmacology and Toxicology, Department of Medical Biology, University of Tromsø - The Arctic University of Norway, Tromsø, Norway.

```
CLUSTAL O(1.2.4) multiple sequence alignment

XP_416204.2_CHICKEN   MRPRVECYSPRFWLVLAVLATTGSGTHAQKSHPSIGIAVILVGTSDEVAIKDAHEKDDFH 60
SP|Q00960|NMDE2_RAT   MKPSAECCSPKFWLVLAVLAVSGSKARSQKSPPSIGIAVILVGTSDEVAIKDAHEKDDFH 60
SP|Q13224|NMDE2_HUMAN MKPRAECCSPKFWLVLAVLAVSGSRARSQKSPPSIGIAVILVGTSDEVAIKDAHEKDDFH 60
                      *:* .** **:*********.:** :::*** ****************************

XP_416204.2_CHICKEN   HLSVVPRVELVAMNETDPKSIITRICDLMSDRKIQGVVFADDTDQEAIAQILDFISAQTL 120
SP|Q00960|NMDE2_RAT   HLSVVPRVELVAMNETDPKSIITRICDLMSDRKIQGVVFADDTDQEAIAQILDFISAQTL 120
SP|Q13224|NMDE2_HUMAN HLSVVPRVELVAMNETDPKSIITRICDLMSDRKIQGVVFADDTDQEAIAQILDFISAQTL 120
                      ************************************************************

XP_416204.2_CHICKEN   TPILGIHGGSSMIMADKDESSMFFQFGPSIEQQASVMLNIMEEYDWYIFSIVTTYFPGYQ 180
SP|Q00960|NMDE2_RAT   TPILGIHGGSSMIMADKDESSMFFQFGPSIEQQASVMLNIMEEYDWYIFSIVTTYFPGYQ 180
SP|Q13224|NMDE2_HUMAN TPILGIHGGSSMIMADKDESSMFFQFGPSIEQQASVMLNIMEEYDWYIFSIVTTYFPGYQ 180
                      ************************************************************

XP_416204.2_CHICKEN   DFVNKIRSTIEHSFVGWELEEVLLLDMSLDDGDSKIQNQLKKLQSPVILLYCTKEEATYI 240
SP|Q00960|NMDE2_RAT   DFVNKIRSTIENSFVGWELEEVLLLDMSLDDGDSKIQNQLKKLQSPIILLYCTKEEATYI 240
SP|Q13224|NMDE2_HUMAN DFVNKIRSTIENSFVGWELEEVLLLDMSLDDGDSKIQNQLKKLQSPIILLYCTKEEATYI 240
                      ***********:**********************************:*************

XP_416204.2_CHICKEN   FEVANSVGLTGYGYTWIVPSLVAGDTDTVPSEFPTGLISVSYDEWDYGLPARVRDGIAII 300
SP|Q00960|NMDE2_RAT   FEVANSVGLTGYGYTWIVPSLVAGDTDTVPSEFPTGLISVSYDEWDYGLPARVRDGIAII 300
SP|Q13224|NMDE2_HUMAN FEVANSVGLTGYGYTWIVPSLVAGDTDTVPAEFPTGLISVSYDEWDYGLPARVRDGIAII 300
                      ******************************:*****************************

XP_416204.2_CHICKEN   TTAASDMLSEHSFIPEPKSSCYNTQEKRIYQSNMLNRYLINVTFEGRNLSFSEDGYQMHP 360
SP|Q00960|NMDE2_RAT   TTAASDMLSEHSFIPEPKSSCYNTHEKRIYQSNMLNRYLINVTFEGRNLSFSEDGYQMHP 360
SP|Q13224|NMDE2_HUMAN TTAASDMLSEHSFIPEPKSSCYNTHEKRIYQSNMLNRYLINVTFEGRNLSFSEDGYQMHP 360
                      ************************:***********************************


XP_416204.2_CHICKEN   KLVIILLTKERKWERVGKWKEKKLQMKYYVWPRFELYPDSEEREDDHLSIVTLEEAPFVI 420
SP|Q00960|NMDE2_RAT   KLVIILLNKERKWERVGKWKDKSLQMKYYVWPRM--CPETEEQEDDHLSIVTLEEAPFVI 418
SP|Q13224|NMDE2_HUMAN KLVIILLNKERKWERVGKWKDKSLQMKYYVWPRM--CPETEEQEDDHLSIVTLEEAPFVI 418
                      *******.************:*.**********:   *::**:*****************
```

```
XP_416204.2_CHICKEN   VENVDPLSGTCMRNTVPCQKRIVTENKTDEEPDYMKKCCKGFCIDILKKISKSVKFTYDL 480
SP|Q00960|NMDE2_RAT   VESVDPLSGTCMRNTVPCQKRIISENKTDEEPGYIKKCCKGFCIDILKKISKSVKFTYDL 478
SP|Q13224|NMDE2_HUMAN VESVDPLSGTCMRNTVPCQKRIVTENKTDEEPGYIKKCCKGFCIDILKKISKSVKFTYDL 478
                      **.*******************::*********.*:************************

XP_416204.2_CHICKEN   YLVTNGKHGKKINGTWNGMIGEVVTKRAYMAVGSLTINEERSEVVDFSVPFIETGISVMV 540
SP|Q00960|NMDE2_RAT   YLVTNGKHGKKINGTWNGMIGEVVMKRAYMAVGSLTINEERSEVVDFSVPFIETGISVMV 538
SP|Q13224|NMDE2_HUMAN YLVTNGKHGKKINGTWNGMIGEVVMKRAYMAVGSLTINEERSEVVDFSVPFIETGISVMV 538
                      ************************ ***********************************

XP_416204.2_CHICKEN   SRSNGTVSPSAFLEPFSADVWVMMFVMLLIISAVAVFVFEYFSPVGYNRCLADGREPGGP 600
SP|Q00960|NMDE2_RAT   SRSNGTVSPSAFLEPFSADVWVMMFVMLLIVSAVAVFVFEYFSPVGYNRCLADGREPGGP 598
SP|Q13224|NMDE2_HUMAN SRSNGTVSPSAFLEPFSADVWVMMFVMLLIVSAVAVFVFEYFSPVGYNRCLADGREPGGP 598
                      ******************************:*****************************

XP_416204.2_CHICKEN   SFTIGKAIWLLWGLVFNNSVPVQNPKGTTSKIMVSVWAFFAVIFLASYTANLAAFMIQEE 660
SP|Q00960|NMDE2_RAT   SFTIGKAIWLLWGLVFNNSVPVQNPKGTTSKIMVSVWAFFAVIFLASYTANLAAFMIQEE 658
SP|Q13224|NMDE2_HUMAN SFTIGKAIWLLWGLVFNNSVPVQNPKGTTSKIMVSVWAFFAVIFLASYTANLAAFMIQEE 658
                      ************************************************************

XP_416204.2_CHICKEN   YVDQVSGLSDKKFQKPNDFSPPFRFGTVPNGSTERNIRNNYPEMHSYMVKFNQRGVDDAL 720
SP|Q00960|NMDE2_RAT   YVDQVSGLSDKKFQRPNDFSPPFRFGTVPNGSTERNIRNNYAEMHAYMGKFNQRGVDDAL 718
SP|Q13224|NMDE2_HUMAN YVDQVSGLSDKKFQRPNDFSPPFRFGTVPNGSTERNIRNNYAEMHAYMGKFNQRGVDDAL 718
                      **************:************************** ***:** ***********

XP_416204.2_CHICKEN   FSLKTGKLDAFIYDAAVLNYMAGRDEGCKLVTIGSGKVFASTGYGIAIQKDSGWKRQVDL 780
SP|Q00960|NMDE2_RAT   LSLKTGKLDAFIYDAAVLNYMAGRDEGCKLVTIGSGKVFASTGYGIAIQKDSGWKRQVDL 778
SP|Q13224|NMDE2_HUMAN LSLKTGKLDAFIYDAAVLNYMAGRDEGCKLVTIGSGKVFASTGYGIAIQKDSGWKRQVDL 778
                      :***********************************************************


XP_416204.2_CHICKEN   AILQLFGDGEMEELEALWLTGICHNEKNEVMSSQLDIDNMAGVFYMLGAAMALSLITFIC 840
SP|Q00960|NMDE2_RAT   AILQLFGDGEMEELEALWLTGICHNEKNEVMSSQLDIDNMAGVFYMLGAAMALSLITFIC 838
SP|Q13224|NMDE2_HUMAN AILQLFGDGEMEELEALWLTGICHNEKNEVMSSQLDIDNMAGVFYMLGAAMALSLITFIC 838
                      ************************************************************
```

```
XP_416204.2_CHICKEN   EHLFYWQFRHCFMGVCSGKPGVVFSISRGIYSCIHGVAIEERQSAMNSPTATMNNTHSNI 900
SP|Q00960|NMDE2_RAT   EHLFYWQFRHCFMGVCSGKPGMVFSISRGIYSCIHGVAIEERQSVMNSPTATMNNTHSNI 898
SP|Q13224|NMDE2_HUMAN EHLFYWQFRHCFMGVCSGKPGMVFSISRGIYSCIHGVAIEERQSVMNSPTATMNNTHSNI 898
                      *********************:**********************.***************

XP_416204.2_CHICKEN   LRLLRTAKNMANLSGVNGSPQSALDFIRRESSVYDISEHRRSFTHSDCKSYNNPPCEENL 960
SP|Q00960|NMDE2_RAT   LRLLRTAKNMANLSGVNGSPQSALDFIRRESSVYDISEHRRSFTHSDCKSYNNPPCEENL 958
SP|Q13224|NMDE2_HUMAN LRLLRTAKNMANLSGVNGSPQSALDFIRRESSVYDISEHRRSFTHSDCKSYNNPPCEENL 958
                      ************************************************************

XP_416204.2_CHICKEN   FSDYISEVERTFGNLQLKDSNVYQDHYHHHHRPHSIGSTSSIDGLYDCDNPPFNAQSRSI 1020
SP|Q00960|NMDE2_RAT   FSDYISEVERTFGNLQLKDSNVYQDHYHHHHRPHSIGSTSSIDGLYDCDNPPFTTQPRSI 1018
SP|Q13224|NMDE2_HUMAN FSDYISEVERTFGNLQLKDSNVYQDHYHHHHRPHSIGSASSIDGLYDCDNPPFTTQSRSI 1018
                      **************************************:**************.:* ***

XP_416204.2_CHICKEN   GKKPLDLGLPPAKHSQLGDLYGKFSFKSDRYGGSGAHDDLIRSDVSDISTHTVTYGNIEG 1080
SP|Q00960|NMDE2_RAT   SKKPLDIGLPSSKHSQLSDLYGKFSFKSDRYSG---HDDLIRSDVSDISTHTVTYGNIEG 1075
SP|Q13224|NMDE2_HUMAN SKKPLDIGLPSSKHSQLSDLYGKFSFKSDRYSG---HDDLIRSDVSDISTHTVTYGNIEG 1075
                      .*****:*** :*****.*************.*   ************************

XP_416204.2_CHICKEN   NAAKRRKQQYKDSLKKRPASAKSRREFDEIELAYRRRPPRSPDHKRYFRDKEGLRDFYLD 1140
SP|Q00960|NMDE2_RAT   NAAKRRKQQYKDSLKKRPASAKSRREFDEIELAYRRRPPRSPDHKRYFRDKEGLRDFYLD 1135
SP|Q13224|NMDE2_HUMAN NAAKRRKQQYKDSLKKRPASAKSRREFDEIELAYRRRPPRSPDHKRYFRDKEGLRDFYLD 1135
                      ************************************************************

XP_416204.2_CHICKEN   QFRAKENSPHWEHVDLTDIYKERGDEFKRDTGGGGGSGSCTNRAHHKHGSGEFGGSNEHK 1200
SP|Q00960|NMDE2_RAT   QFRTKENSPHWEHVDLTDIYKERSDDFKRDSVSGG--GPCTNRSHLKHGTGE-------K 1186
SP|Q13224|NMDE2_HUMAN QFRTKENSPHWEHVDLTDIYKERSDDFKRDSVSGG--GPCTNRSHIKHGTGD-------K 1186
                      ***:*******************.*:****: .**  * ****:* ***:*:       *

XP_416204.2_CHICKEN   HGVVSGVPAPWEKNLTNLDWEDRSGANFCRSCPSKVHNYTPSVVGQNSTRQACIRCEACK 1260
SP|Q00960|NMDE2_RAT   HGVVGGVPAPWEKNLTNVDWEDRSGGNFCRSCPSKLHNYSSTVAGQNSGRQACIRCEACK 1246
SP|Q13224|NMDE2_HUMAN HGVVSGVPAPWEKNLTNVEWEDRSGGNFCRSCPSKLHNYSTTVTGQNSGRQACIRCEACK 1246
                      ****.************::******.*********:***: :*.**** ***********

XP_416204.2_CHICKEN   KAGNLYDISEDNSLQELDQPPAPVPVATSATSSSKYPQSPSNSASKVQKKNRNKLRRQHS 1320
SP|Q00960|NMDE2_RAT   KAGNLYDISEDNSLQELDQPAAPVAVTSN-ASSTKYPQSPTNS--KAQKKNRNKLRRQHS 1303
SP|Q13224|NMDE2_HUMAN KAGNLYDISEDNSLQELDQPAAPVAVTSN-ASTTKYPQSPTNS--KAQKKNRNKLRRQHS 1303
                      ******************** *** *::. :*::******:**  *.*************
```

```
XP_416204.2_CHICKEN   YDTFVDLQKDDSALAPRSVSLKDKGRFLEGSPYAHMFEMPASETTFANNKSSVPATSYHH 1380
SP|Q00960|NMDE2_RAT   YDTFVDLQKEEAALAPRSVSLKDKGRFMDGSPYAHMFEMPAGESSFAN-KSSVPTAGH-H 1361
SP|Q13224|NMDE2_HUMAN YDTFVDLQKEEAALAPRSVSLKDKGRFMDGSPYAHMFEMSAGESTFANNKSSVPTAGHHH 1363
                      *********:::***************::********** *.*::*** *****::.: *

XP_416204.2_CHICKEN   HNNPGSSGGYMLSKSLYPDRVTQNPFIPTFGDDQCLLHGSKSYFFRQPVVAGGPKARPDF 1440
SP|Q00960|NMDE2_RAT   HNNPG--SGYMLSKSLYPDRVTQNPFIPTFGDDQCLLHGSKSYFFRQPTVAGASKTRPDF 1419
SP|Q13224|NMDE2_HUMAN HNNPG--GGYMLSKSLYPDRVTQNPFIPTFGDDQCLLHGSKSYFFRQPTVAGASKARPDF 1421
                      *****  .****************************************.***. *:****

XP_416204.2_CHICKEN   RAIVTTNKPVVSALHGAVPARFQKDICIGNQSNPCVPNNKNPRAFNGSSNGHVYEKLSSI 1500
SP|Q00960|NMDE2_RAT   RALV-TNKPVVVTLHGAVPGRFQKDICIGNQSNPCVPNNKNPRAFNGSSNGHVYEKLSSI 1478
SP|Q13224|NMDE2_HUMAN RALV-TNKPVVSALHGAVPARFQKDICIGNQSNPCVPNNKNPRAFNGSSNGHVYEKLSSI 1480
                      **:* ****** :******.***************************************

XP_416204.2_CHICKEN   ESDV 1504
SP|Q00960|NMDE2_RAT   ESDV 1482
SP|Q13224|NMDE2_HUMAN ESDV 1484
                      ****
```

Figure S-1: Alignment of the amino acid sequence of chicken, rat and human GluN2B subunits, using Clustal Omega multiple sequence alignment program. Identical amino acids are indicated with an asterisk (*), non-identical amino acid with weakly similar properties is denoted with a period (.), while amino acids with strongly similar properties are marked with a colon(:). Gaps in the sequences are shown with hyphens (-) and non-similar amino acids are indicated with an open space ( ). The total rat and human GluN2B amino acid sequence is 93% identical to the chicken GluN2B sequence. The rat and human GluN2B sequences are 98% similar to each other.

A

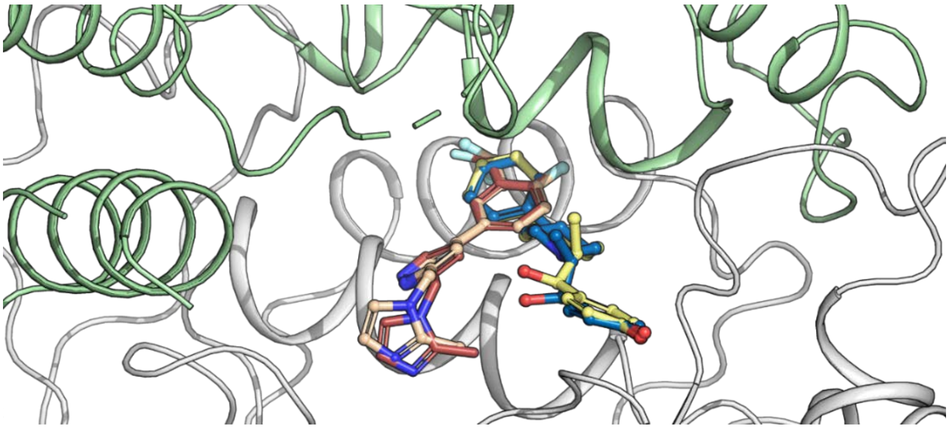

B

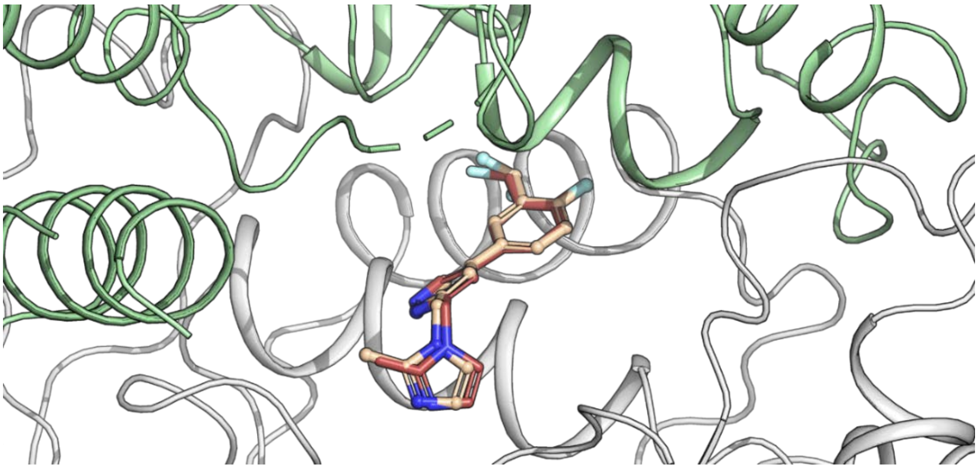

Figure S-2: Comparison of the binding poses of ifenprodil and EVT-101 in their respective experimental structure versus their docking pose in the generated chicken binding pocket. The receptor is showed as a ribbon structure with the GluN1 subunit chain colored pale green and GluN2B subunit chain in grey. The ligands are depicted as stick structures and residues 127-136 of GluN1A are hidden for better clarity. **A:** ATD domain in complex with ifenprodil (PDB id: 5EWJ), with the experimental pose of ifenprodil in metallic blue and the chicken docking pose showed in yellow (glide score: -10.73 kcal/mol). The EVT-101 binding pose in the experimental structure (dimer A) shown in red, while its docking pose in the chicken binding pocket is shown in beige (glide score: -10.62 kcal/mol). **B:** Alternative conformation of EVT-101 found in the B dimer and its docking pose (glide score: -10.62 kcal/mol).

A

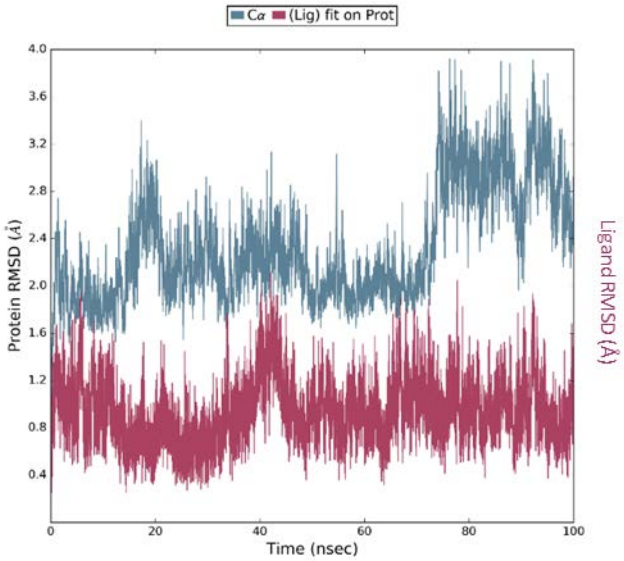

Cα
(Lig) fit on Prot
4.0
3.6
3.2
2.8
2.4
2.0
1.6
1.2
0.8
0.4
Protein RMSD (Å)
Ligand RMSD (Å)
0
20
40
60
80
100
Time (nsec)

B

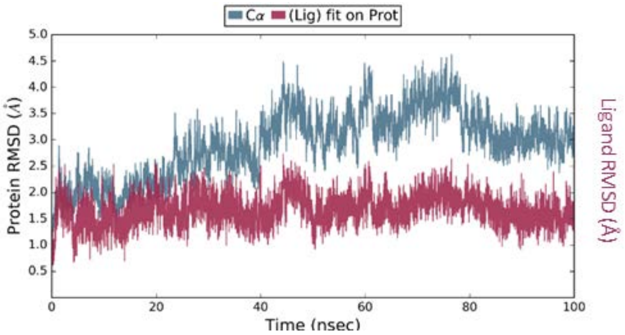

Cα
(Lig) fit on Prot
5.0
4.5
4.0
3.5
3.0
2.5
2.0
1.5
1.0
0.5
Protein RMSD (Å)
Ligand RMSD (Å)
0
20
40
60
80
100
Time (nsec)

C

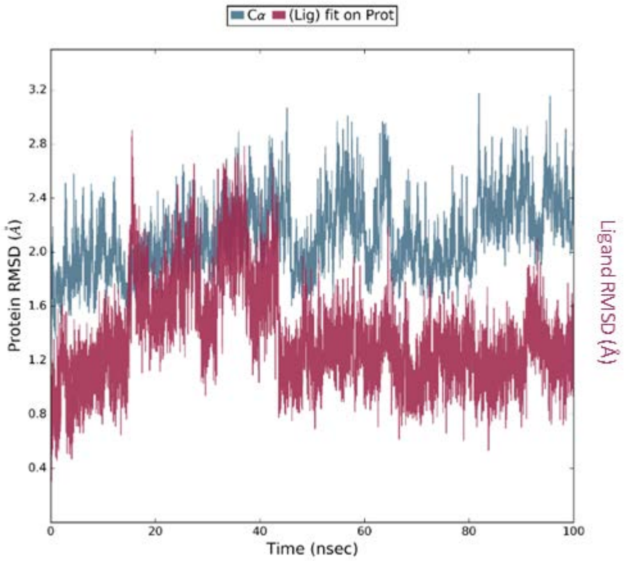

Cα
(Lig) fit on Prot
3.2
2.8
2.4
2.0
1.6
1.2
0.8
0.4
Protein RMSD (Å)
Ligand RMSD (Å)
0
20
40
60
80
100
Time (nsec)

D

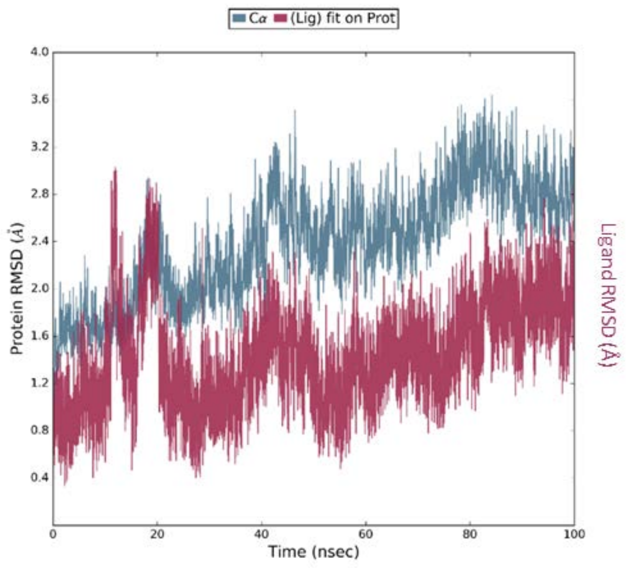

E

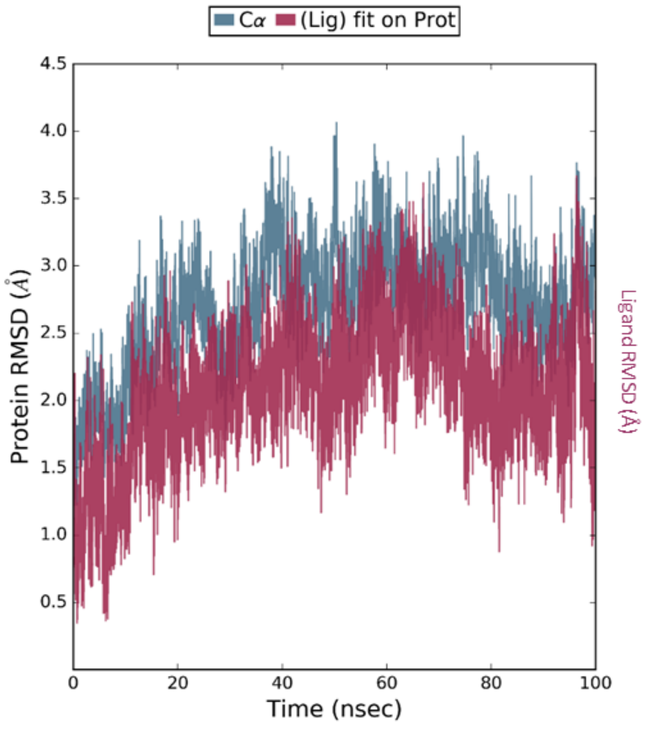

Figure S-3: Protein and Ligand RMDS. RMSD evolution of the binding site and ligand during simulation (0-100 ns). A: Ro 25-6981, B: eliprodil, C: ifenprodil, D: EVT-101, E: Ro 25-6981.

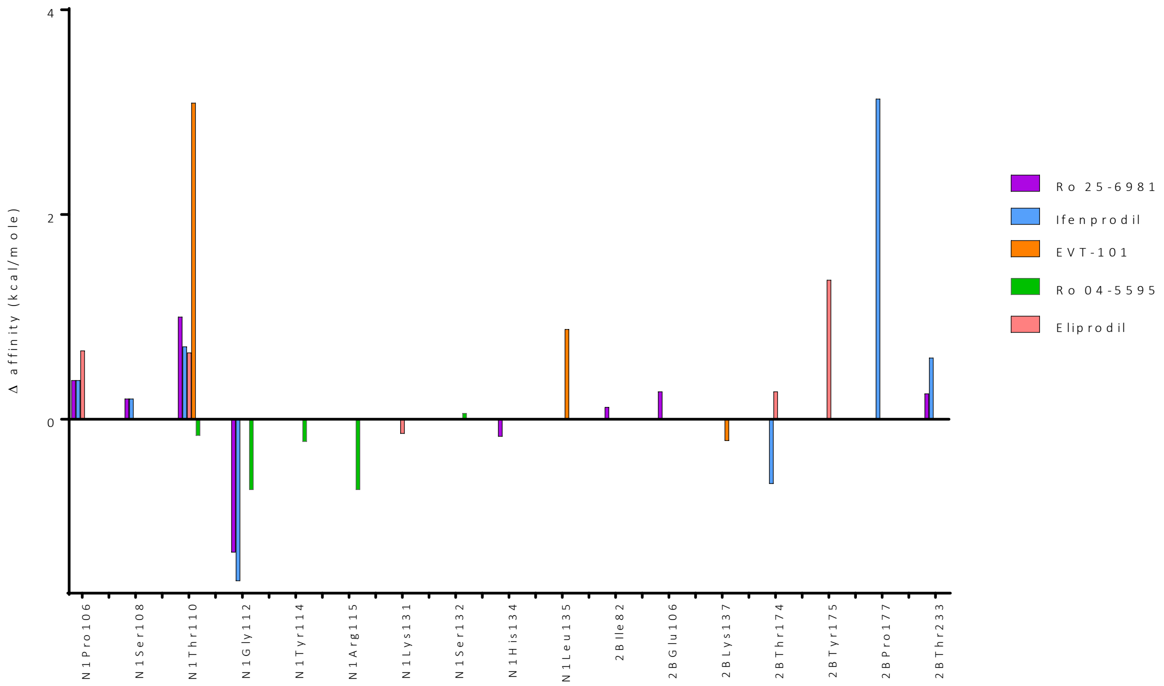

Figure S-4: Overview of Δ affinities after alanine mutation scanning on residues not predicted to interact directly with the ligands during the last 10 ns of simulation. Prefix N1 denotes that the residue is located in the GluN1 subunit, while 2B indicates the GluN2B subunit. These residues affects the affinity of the ligands, probably by being important for the conformation of the binding pocket.

A

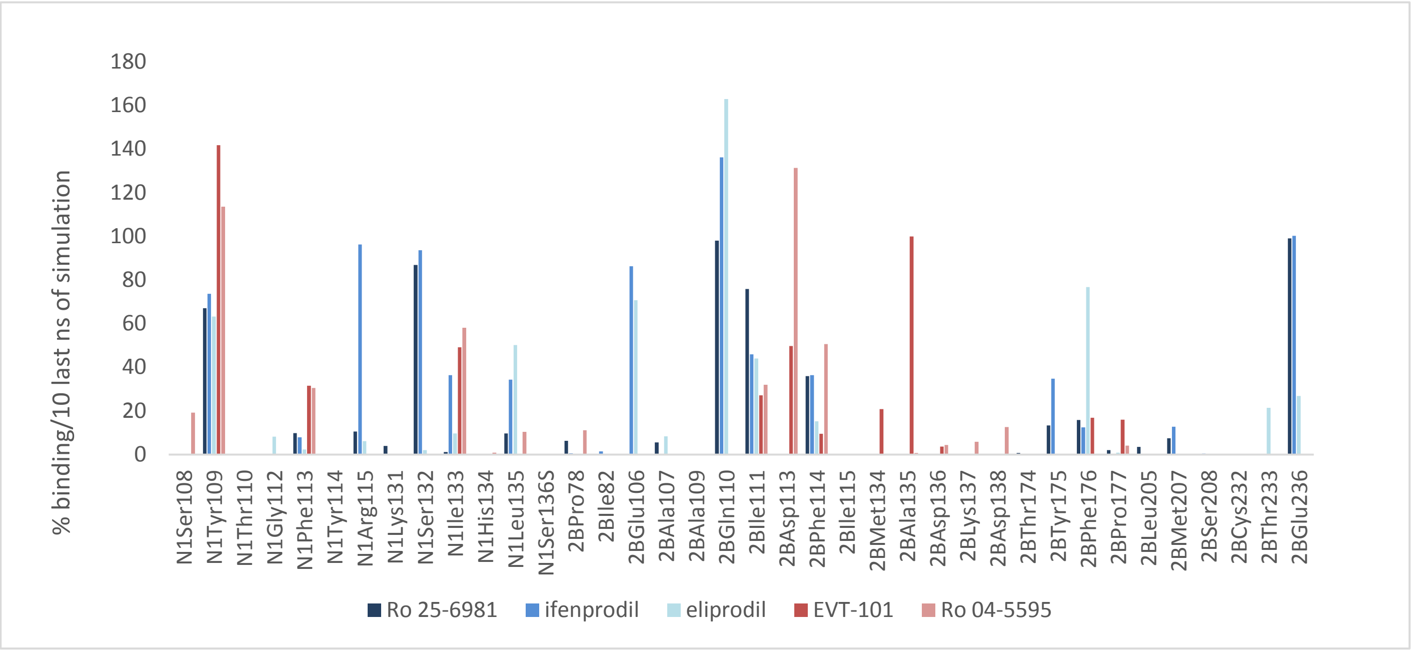

B

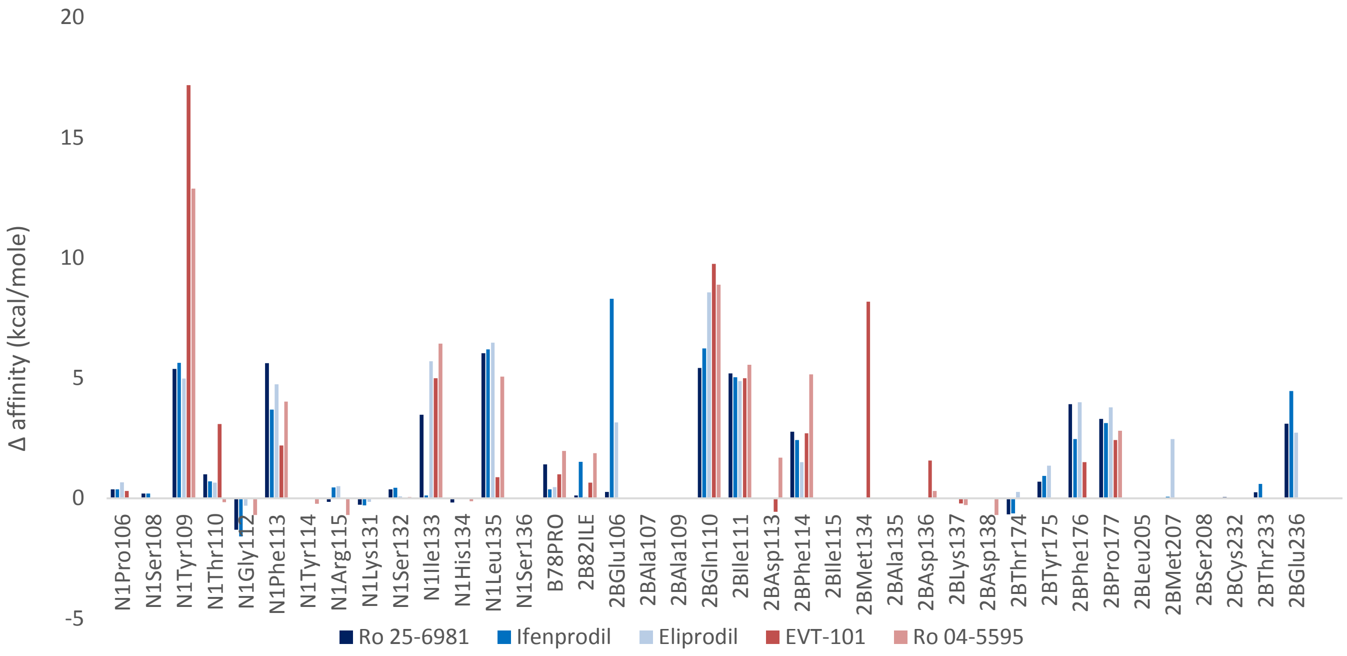

Figure S-5: Overview of the ligand interaction percentage and Δ affinities after alanine mutation scanning. Prefix N1 denotes that the residue is located in the GluN1 subunit, while 2B indicates the GluN2B subunit. The ligand interaction percentage is given as percent interaction during the last 10 ns of simulation (A), and alanine scanning calculation was performed on receptor-ligand complexes averaged over the last 10 ns (B).
